# Genome scale transcriptional response diversity among ten ecotypes of *Arabidopsis thaliana* during heat stress

**DOI:** 10.3389/fpls.2013.00532

**Published:** 2013-12-26

**Authors:** Pankaj Barah, Naresh D. Jayavelu, John Mundy, Atle M. Bones

**Affiliations:** ^1^Cell Molecular Biology and Genomics Group, Department of Biology, Norwegian University of Science and TechnologyTrondheim, Norway; ^2^Department of Chemical Engineering, Norwegian University of Science and TechnologyTrondheim, Norway; ^3^Department of Biology, University of CopenhagenCopenhagen, Denmark

**Keywords:** heat stress, natural variation, microarray transcriptional profiling, regulatory networks, systems biology, *Arabidopsis thaliana*, ecotypes

## Abstract

In the scenario of global warming and climate change, heat stress is a serious threat to crop production worldwide. Being sessile, plants cannot escape from heat. Plants have developed various adaptive mechanisms to survive heat stress. Several studies have focused on diversity of heat tolerance levels in divergent *Arabidopsis thaliana (A. thaliana)* ecotypes, but comprehensive genome scale understanding of heat stress response in plants is still lacking. Here we report the genome scale transcript responses to heat stress of 10 *A. thaliana* ecotypes (Col, L*er*, C24, Cvi, Kas1, An1, Sha, Kyo2, Eri, and Kond) originated from different geographical locations. During the experiment, *A. thaliana* plants were subjected to heat stress (38°C) and transcript responses were monitored using Arabidopsis NimbleGen ATH6 microarrays. The responses of *A. thaliana* ecotypes exhibited considerable variation in the transcript abundance levels. In total, 3644 transcripts were significantly heat regulated (*p* < 0.01) in the 10 ecotypes, including 244 transcription factors and 203 transposable elements. By employing a systems genetics approach- Network Component Analysis (NCA), we have constructed an *in silico* transcript regulatory network model for 35 heat responsive transcription factors during cellular responses to heat stress in *A. thaliana*. The computed activities of the 35 transcription factors showed ecotype specific responses to the heat treatment.

## Introduction

Climate change is increasingly viewed as a current and future cause of hunger and poverty (Lobell et al., 2011; Wheeler and von Braun, 2013). In the scenario of global climatic change, different environmental stresses are severe threats to agricultural production worldwide (Brown and Funk, 2008; Ahuja et al., 2010). Among all stress conditions, elevated temperature is seen as the most serious threat to crop production (Wheeler et al., 2000; Ciais et al., 2005; Semenov and Shewry, 2011). Recurrent heat stress also affects disease resistance in plants by suppressing plant immunity, as plant heat stress and defense responses share important mediators such as calcium ions and heat shock proteins (HSPs) (Lee et al., 2012). Climate data suggest that heat waves became more common during the twentieth century (Stott et al., 2004). Recently, Bita et al. reviewed the effects of high temperature stress on physiology, biochemistry, and gene regulation pathways in plants leading to catastrophic loss of crop productivity (Bita and Gerats, 2013). Transient or continuous high temperatures cause a range of morphological, physiological, and biochemical changes in plants which affect growth and development and may lead to a drastic reduction in economic yield (Richter et al., 2010). Plants are highly sensitive to temperature and can differentiate minute variations of as little as 1°C (Mittler et al., 2012). Upon exposure to heat stress, seed germination, and photosynthetic efficiency decline (Endo et al., 2009). Considering the predicted severity of changing climatic situation, dissecting the molecular basis of heat stress responses in plants, and identifying key components of the heat stress sensing and signal transduction pathways, are becoming major concern of present time (Bita and Gerats, 2013; Qu et al., 2013). Such knowledge could be used toward developing plants and crops with enhanced tolerance to heat stress (Zhang et al., 2000; Mittler and Blumwald, 2010).

Environmental stress is a key factor driving the genome regulation, evolutionary history, and geographical distribution of organisms including plants (Alonso-Blanco et al., 2009). Intraspecific natural variation or within-species phenotypic variation caused by spontaneous, favorable mutations contribute to the local adaptations of plants (Mitchell-Olds and Schmitt, 2006). Such natural variation in crop plants has been exploited by human society for the selection of developmental traits and physiological features beneficial for agriculture (Weigel and Nordborg, 2005; Doebley et al., 2006). Additionally, studying natural variation in wild species can tell us about the molecular basis of phenotypic differences related to plant adaptation to diverse natural environments (Borevitz and Nordborg, 2003). There have been very few studies conducted till date focusing on the diversity of heat tolerance in phenotypically divergent ecotypes (Alonso-Blanco and Koornneef, 2000; Larkindale et al., 2005; Al-Quraan et al., 2012). Thus, the molecular basis of the natural variation during heat stress response in plants at genome scale is not fully understood yet (Yeh et al., 2012).

Transcriptomics, proteomics and metabolomics approaches have been frequently used to identify heat stress-responsive genes, proteins, and metabolites in plants (Kaplan et al., 2004; Jagadish et al., 2010; Pecinka et al., 2010; Weston et al., 2011; Zou et al., 2011b; Rocco et al., 2013). Transcript profiling is a major tool to identify genes exhibiting transcriptional regulation in response to changing environmental conditions. For such studies in plants, *A. thaliana* remains a model system (Somerville and Koornneef, 2002). Variation in experimental conditions and protocols makes it difficult to extract and compare information from data sets produced by individual laboratories (Moreau et al., 2003). To overcome such problems, 10 ecotypes of *A. thaliana* were subjected to 5 individual stress treatments and 6 combinations of these stress treatments under the same experimental set up and profiling protocols (Rasmussen et al., 2013). We have considered all the heat experiments conducted on 10 ecotypes from this published dataset (GEO accession**GSE41935**) to explore genome-scale transcriptomic response signatures of *A. thaliana* during heat stress treatment. Being highly dynamic in nature, any biological system changes in response to environmental and genetic perturbations. Differential dynamic network mapping facilitates the exploration of previously unknown interactions (Ideker and Krogan, 2012). While the *A. thaliana* genome has ~1922 TFs (Guo et al., 2005), experimentally confirmed regulatory relations are available for less than 100 TFs, as per information from the AGRIS database version updated in September, 2012 (Davuluri et al., 2003). Tirosh et al. (Tirosh and Barkai, 2011) have explained how regulatory relationships can also be deduced from patterns of evolutionary divergence in molecular properties such as gene expression (Keurentjes et al., 2007). To compensate the lack of information on transcription factor activity at the genome-scale, computational algorithms have been developed to identify regulatory modules and their condition-specific regulators from gene expression data (Alter et al., 2000; Segal et al., 2003; Herrgard et al., 2004). Network Component Analysis (NCA) is such an approach, which has been successfully implemented in species including *A. thaliana* to determine both the activities and regulatory influences for a set of transcription factors on target genes (Liao et al., 2003; Kao et al., 2004; Wang et al., 2011). Using the NCA method, we have predicted ecotype specific regulatory relationships which generated new information toward understanding the natural variation in heat response pattern among different ecotypes of the model plant *A. thaliana.*

## Results

### Different transcriptome signatures of 10 A*rabidopsis* ecotypes responding to heat stress

**Figure 1:**
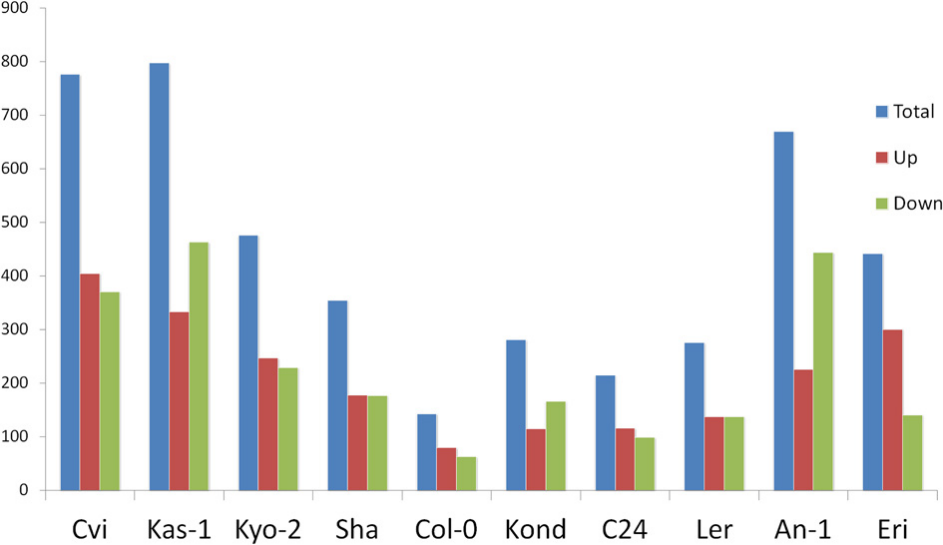
**Numbers of differentially regulated transcripts in each of the 10 ecotypes at significance level *p* ≤ 0.01**. Ecotypes are on the x axis and numbers of differentially regulated transcripts on the y axis. Blue bar represents total number of differentially regulated transcripts, red bar the number of positively regulated (up) transcripts and green bar represents number of negatively regulated (down) transcripts.

**Figure 2:**
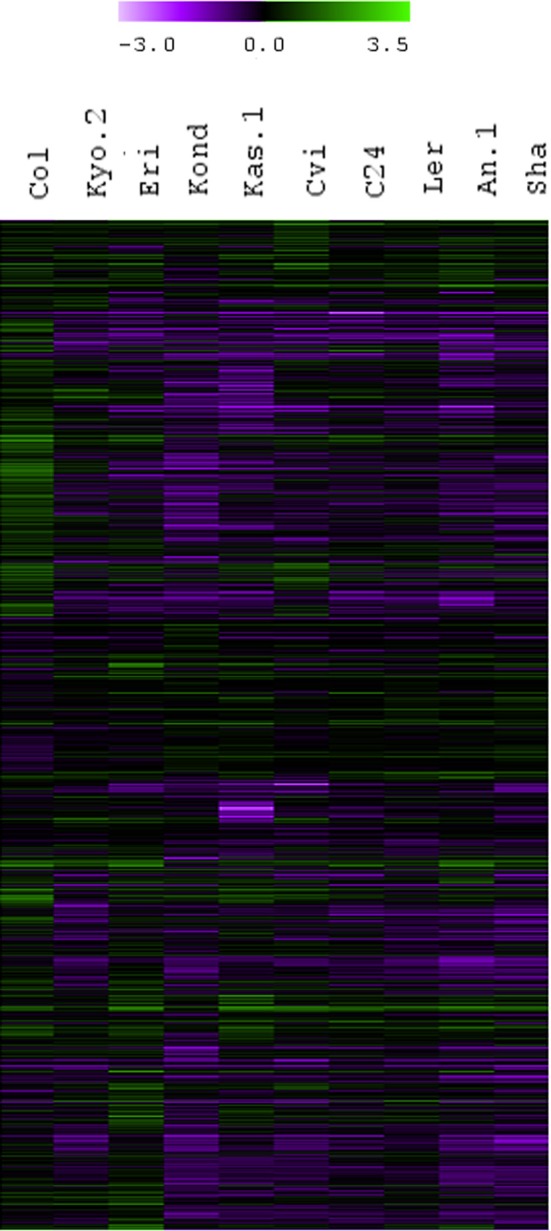
**Fold change values (treatment vs. control) calculated from normalized expression index for top 1000 significant genes from all the 10 ecotypes.** Hierarchical clustering (HCL) was performed with Pearson correlation using average linkage method and 10,000 bootstrapping for the top 1000 heat regulated transcripts based on fold-change ratios compared to their respective controls.

To cover a wide array of phenotypic variations, 10 natural accessions of *A. thaliana* representing their originally reported habitats from 16 to 56.5° north latitudes were selected during the ERA-PG Multi-stress project. These accessions or ecotypes were- Cvi (Cape Verde Islands), Kas-1 (Kashmir, India), Kyo-2 (Kyoto, Japan), Sha (Shakdara, Tadjikistan), Col-0(Columbia, USA), Kond (Kondara, Tadjikistan), C24 (Coimbra, Portugal), L*er* (Landsberg, Poland), An-1 (Antwerpe, Belgium), Eri (Erigsboda, Sweden) (details in Table 1). We chose a cut-off *p* ≤ 0.01 to define a gene as differentially stress regulated. Using the results from the 10 ecotypes, we examined the differences in transcript abundences that occurred during early hours of heat treatment (38°C). The results (Table 1 and Figure 1) indicated that the *A. thaliana* ecotypes have visibly different transcriptome level signatures in response to heat stress. Variable numbers of transcripts were up or down regulated among the ecotypes (Table 1). Kas-1 (797) and Cvi (776) exhibited higher numbers of differentially regulated transcripts while Col-0 (143) had comparatively few differentially regulated transcripts. A unified list of 3644 differentially regulated transcripts (*p* < 0.01) was generated from the 10 ecotypes (Table S1A.) Surprisingly, 3114 (85%) transcripts were differentially regulated in only one of the 10 ecotypes. Figure 2 displays fold change values (treatment vs. control) calculated from normalized expression index for the top 1000 most significant genes from the 10 ecotypes. Global observation of the heat map indicates differentially regulated transcriptome signatures in response to heat treatment in the 10 ecotypes. The significant list of differentially regulated transcripts includes most of the previously documented heat regulated genes including *Hsps* (heat shock proteins) and *Hsfs* (heat shock transcription factors) (Swindell et al., 2007).

**Table 1:** **Summary of the ecotypes and their gene expression pattern during heat stress**.

**Eotype**	*Geographic origin	**Latitude (°North)**	**Total**	**Total up**	**Total down**	**Unique (total)**	**Unique (up)**	**Unique (down)**
Cvi	Cape Verdia Islands	16	776	405	371	649	348	301
Kas-1	Kashmir, India	34	797	334	463	569	219	350
Kyo-2	Kyoto city, western part of Hoshu Island, Japan	35.5	476	247	229	324	159	165
Sha	Shakdara, Pamiro-Alay, Tadjikistan	39	355	178	177	206	92	114
Col-0	Columbia, United States	38.5	143	80	63	105	56	49
Kond	Kondara, Tadjikistan	38.8	281	115	166	183	72	111
C24	Coimbra, Portugal	40	215	116	99	115	60	55
L*er*	Landsberg, Poland	48	276	138	138	224	113	111
An-1	Antwerpern, Belgium	51.5	670	226	444	450	137	313
Eri	Erigsboda, Sweden	56	442	301	141	290	193	97

* Geographic origins of the ecotypes were collected from the donor, TAIR and the Arabidopsis 1001 Genome project database.

Geographic distribution of the 10 A. thaliana ecotypes and number of heat regulated genes in each of the ecotypes (*p* ≤ 0.01). Up and down regulation was calculated based on fold change ratios compared to respective untreated controls in individual ecotypes. (Unique, Unique to the respective ecotype).

### Ecotype specific heat regulated transcript lists contain many transcription factors (TFs) and transposable elements (TEs)

The unified list of 3644 differentially regulated transcripts during the heat stress contained 244 TFs (annotated in Table S1B). Only AT5G57660 (*CONSTANS-like 5 zinc finger family protein*) was significantly (*p* ≤ 0.01) upregulated in all of the 10 ecotypes. Two other TFs, AT4G25480 (Dehydration response element B1A) and AT5G24470 (Arabidopsis pseudo-response regulator 5), were significantly upregulated in 9 ecotypes. *MBF1C*/AT3G24500 (multiprotein bridging factor 1C) was significantly down-regulated in 8 ecotypes (Suzuki et al., 2008). Among others, 70 TFs were significantly regulated in 2 ecotypes and 62, TFs were significantly regulated only in one of the ecotypes. The differentially regulated TFs included members of prominent TF families such as *ABF3,ADOF, AFO, AGL, NAC, AP1, AP2, Prr5, ARF, bZIP, HSF, IDD, MYB, BLJ, DNAJ, JAZ, MYB, PHD finger, WRKY, C2H2 zinc finger* etc. Such differential regulation of diverse TF families was obvious from the fact that apart from heat shock protein induction, other pathways involving ethylene, salicylic acid (*SA*), and trehalose were shown to play crucial roles in plant thermotolerance (Larkindale and Knight, 2002; Larkindale et al., 2005).

The Nimblgen12-plex Arabidopsis microarray chip included 3822 transposable element (TE) probes. Of them, 203 TEs were differentially regulated during heat stress (Table S1C). Except for 5, TEs, the rest were differentially regulated in single ecotypes. The distribution of the differentially regulated TEs in ten ecotypes were: Col-0 (10), L*er* (24), Cvi (24), Eri (18), Kas2 (27), Kond (23), Kyo2 (30), C24 (11), Sha (27), and An1 (14).

### Gene set enrichment analysis (GSEA) indicates activation of diverse processes

To investigate functionally over-represented gene ontology categories, BinGO software was used on the list of 3644 differentially regulated transcripts from the 10 ecotypes. No annotations were retrieved for 60 genes which were eliminated from the final analysis. In total, 82 statistically significant gene ontology categories were detected, including many parent categories such as response to stimulus, stress, biotic stimulus, abiotic stimulus etc. (Table S2). Apart from these global terms, genes showing significant variation in mRNA levels in *A. thaliana* during heat stress were mainly belong to categories like response to heat, temperature stimulus, water deprivation, light stimulus, wounding, osmotic stress, oxidative stress, salt stress, and protein folding etc. The rest of the differentially regulated genes covered various functions, such as transcription, translation, signaling, metabolism, and general stress response. These results indicated that, during exposure to heat stress, plants extensively reprogrammed gene expression, to limit damage caused by high temperatures.

### *Hsp* genes exhibit differential expression patterns in *arabidopsis* ecotypes during heat stress

**Figure 3:**
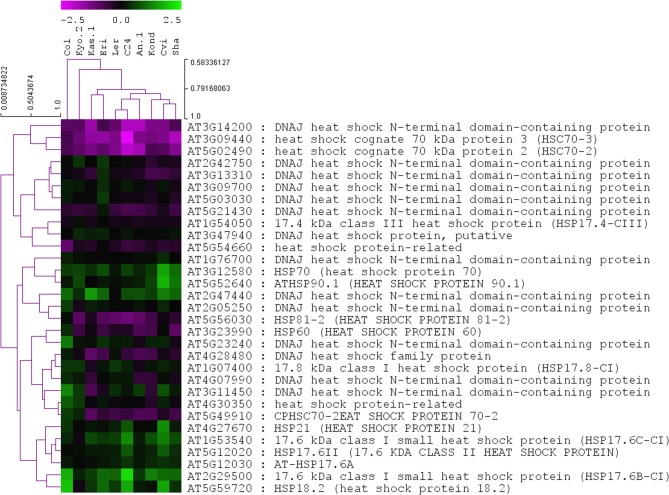
**Heat map of log2 fold change values of the 31 significantly regulated (*p* ≤ 0.01) *HSP* genes in 10 *A. thaliana* ecotypes.** Genes and ecotypes were clustered using Pearson's correlation coefficient with average linkage method. The *P*-values and log2 fold-change values associated with all of the 145 *Hsp*s are provided in Table S3.

A list of total 145 *Hsps* was generated containing the term “heat shock protein” as per annotations available from TAIR10 database (Table S3). Among them, 31 *Hsps* were significantly (*p* = 0.01) differentially regulated in at least one of the 10 ecotypes. Most of them were encoded for DNAJ heat shock N-terminal domain-containing proteins. Other upregulated members were *HSP70, HSP21, HSP17, HSP18* etc. None of the 31 significant *HSPs* were expressed in a similar pattern across all 10 ecotypes, which indicated differentially regulated activity profiles of them across *A. thaliana* ecotypes during heat stress responses (Figure 3).

### Re-construction of a transcriptional regulatory network during the heat stress response in *A. thaliana*

By looking at the differential expression levels of a large number of TFs during the heat experiments, we wanted to explore the pattern of regulatory interactions between the TFs and their corresponding target genes (TGs) in the 10 *A. thaliana* ecotypes using a benchmarked algorithm, NCA (Liao et al., 2003; Wang et al., 2011; Barah et al., 2013). Simple correlation between the expression profile of a transcription factor and its targets is not obvious, and simple clustering based methods have not been very successful in deciphering them (Qian et al., 2003). The key assumption during predictions of interactions between TFs and their target genes using gene expression data is that high dimensional mRNA expression profiles contain hidden regulatory signals which can be decomposed to low-dimensional regulatory signals driven through an interacting network (Holter et al., 2000; Carrera et al., 2009). The lower dimensional regulatory signals can be represented as a bipartite networked system of the transcription factors and the target genes in which the gene expression levels are transformed into weighted functions of the intracellular states corresponding to the activity of the transcription factors.

**Figure 4:**
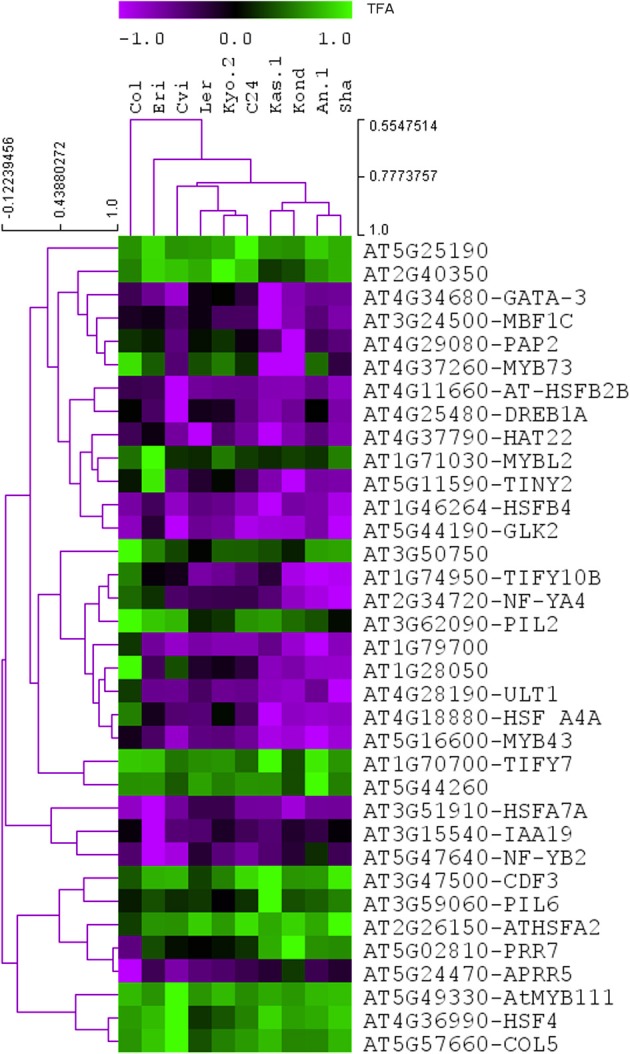
**Predicted the activities of the 35 TFs among the 10 ecotypes.** The NCA algorithm predicts the activities of the TFs based on differential expression profiles (log2 fold change values) of their linked TGs. The predicted activity profiles of the 35 heat regulated TFs shows variations in the 10 *A. thaliana* ecotypes.

The NCA algorithm requires two inputs to calculate the hidden regulatory activity profiles: a series of gene expression profiles and a pre-defined regulatory network. A list of 1922, TFs in *A. thaliana* genome were collected from the Database of Arabidopsis Transcription Factors (DATF) (Guo et al., 2005), The Arabidopsis Gene Regulatory Information Server (AGRIS) (Yilmaz et al., 2011), and the Plant Transcription Factor Database (PlantTFDB) (Riano-Pachon et al., 2007). A list of 59 previously known heat regulated transcription factors was generated from the Gene Ontology database (Ashburner et al., 2000) under the annotation category “response to heat” or containing the term “heat shock factor.” The list of differentially regulated TFs in our transcriptome data contains 35 out the curated list of 59 heat responsive factors. A bipartite co-regulatory network (Alvarez and Woolf, 2011) was constructed from the gene expression values based on correlation-coefficient threshold ≥ 0.8 between the 35 heat regulated TFs and 1294, TGs (Table S4). The resulting network contained 1947 connections. Of them, 687 connections were activations (positive) and 1260 were repressions (negative). Few of the TFs in the network are highly connected (hubs), which supports the scale-free behavior of the predicted TF-TG network (Albert, 2005). The number of connections for each of the TFs is listed in Table 2. This co-regulatory network model was further used as an input to the NCA algorithm to predict the activities of the TFs based on differential expression profiles (log2 fold change values) of their linked TGs (Figure 4). Noticeable variation was observed in the activity profiles of the 35 TFs among the 10 ecotypes.

**Table 2:** **Number of predicted regulatory connections for each of the TFs**.

**TAIR locus**	**Short annotations**	**Number of connections**	**Activations**	**Repressions**
AT1G74950	*TIFY10B*	258	238	20
AT4G11660	*HSFB2B*	182	21	161
AT1G28050	*AT1G28050*	149	120	29
AT5G49330	*MYB111*	131	114	17
AT5G16600	*MYB43*	123	79	44
AT5G47640	*NF-YB2*	120	97	23
AT5G44260	*AT5G44260*	99	36	63
AT5G57660	*COL5*	81	23	58
AT4G18880	*HSF A4A*	79	52	27
AT1G46264	*HSFB4*	67	61	6
AT3G24500	*MBF1C*	58	49	9
AT2G34720	*NF-YA4*	53	33	20
AT1G79700	*AT1G79700*	52	14	38
AT5G11590	*TINY2*	51	41	10
AT4G25480	*DREB1A*	49	37	12
AT5G44190	*GLK2*	47	37	10
AT5G02810	*PRR7*	36	28	8
AT5G24470	*APRR5*	35	23	12
AT4G34680	*GATA-3*	34	27	7
AT5G25190	*AT5G25190*	25	11	14
AT4G28190	*ULT1*	24	11	13
AT4G36990	*HSF4*	21	6	15
AT4G37260	*MYB73*	21	11	10
AT3G15540	*IAA19*	20	15	5
AT1G70700	*TIFY7*	17	10	7
AT2G40350	*AT2G40350*	15	7	8
AT3G51910	*HSFA7A*	15	10	5
AT3G62090	*PIL2*	14	6	8
AT4G29080	*PAP2*	14	13	1
AT3G50750	*AT3G50750*	12	2	10
AT3G59060	*PIL6*	11	7	4
AT3G47500	*CDF3*	10	3	7
AT1G71030	*MYBL2*	9	8	1
AT2G26150	*HSFA2*	9	6	3
AT4G37790	*HAT22*	6	4	2

Few TFs have higher connections than others supporting the scale-free behavior of the predicted TF-TG network. Activations and repressions are calculated based on positive and negative correlations, respectively.

The predicted activity profiles of the 35 heat regulated TFs clearly show the ecotype specific activities in the 10 *A. thaliana* ecotypes. For example, transcription factor AT5G02810 (*PRR7*) was highly active in the Kond ecotype. We identified both multi responsive (active in more than one ecotype) and unique responsive transcription factors (active only in one specific ecotype). The detailed results are provided in Table 3. The majority of the ecotype specific transcription factors were active in Cvi ecotype in response to heat treatment. Multi responsive transcription factors are mostly active in Kond, An-1 and Sha. The transcription factor AT1G74950 (*TIFY10B*) is highly responsive in Kond, An-1 and Sha.

**Table 3:** **Ecotype specific transcriptional activity profiles of the 35 heat responsive TFs**.

**TF ID**	**Alias**	**Ecotypes**
AT1G74950	*TIFY10B*	Kond, An-1, Sha
AT4G11660	*HSFB2B*	Cvi
AT1G28050	*AT1G28050*	Kyo-2, An-1, Col, Sha
AT5G49330	*MYB111*	Cvi
AT5G16600	*MYB43*	Kas-1, Kond, An-1, Sha
AT5G47640	*NF-YB2*	Cvi, Eri,
AT5G44260	*AT5G44260*	An-1
AT5G57660	*COL5*	Cvi
AT4G18880	*HSF A4A*	Kas-1, An-1
AT1G46264	*HSFB4*	Kas-1, Sha
AT3G24500	*MBF1C*	Kas-1, Eri
AT2G34720	*NF-YA4*	An-1, Sha
AT1G79700	*AT1G79700*	Kond, An-1
AT5G11590	*TINY2*	Eri, Kond, Col
AT4G25480	*DREB1A*	Cvi
AT5G44190	*GLK2*	Cvi, Kas-1
AT5G02810	*PRR7*	Kond
AT5G24470	*APRR5*	Col
AT4G34680	*GATA-3*	Cvi, Kas-1
AT5G25190	*AT5G25190*	Eri, Kond, C24, An-1
AT4G28190	*ULT1*	Kond, Sha
AT4G36990	*HSF4*	Cvi, Kas-1, Sha
AT4G37260	*MYB73*	Kas-1, Kond, Col
AT3G15540	*IAA19*	Eri
AT1G70700	*TIFY7*	Kas-1, An-1
AT2G40350	*AT2G40350*	Kyo-2, Eri
AT3G51910	*HSFA7A*	Eri, Kond
AT3G62090	*PIL2*	Col
AT4G29080	*PAP2*	Kond
AT3G50750	*AT3G50750*	Col
AT3G59060	*PIL6*	Kas-1
AT3G47500	*CDF3*	Kas-1, C24, Sha
AT1G71030	*MYBL2*	Eri
AT2G26150	*ATHSFA2*	Ler, Kond, C24, Sha
AT4G37790	*HAT22*	Ler, Kas-1

This table presents, which of the 35 previously reported heat responsive TFs are active among 10 ecotypes during our experiments based on their predicted activity profiles using NCA algorithm.

## Discussion

Here we undertook an experiment to analyze the natural variation in genome-scale heat stress response in 10 *A. thaliana* ecotypes at a single time point (3 h) of gene expression measurement. The analysis indicated that the 10 *A. thaliana* ecotypes had significantly different transcriptome level signatures in response to heat stress. It raises question about global acceptability of results generated from previously conducted plant stress experiments based only on Col-0 and L*er* as model ecotypes.

Among the differentially heat regulated transcripts, 85% showed ecotype specific expression patterns. Heat shock proteins or molecular chaperones were the most prominent group within the list of differentially regulated list of transcripts. Apart from common, heat stress related functional categories, GSEA of the differentially regulated transcripts uncovered many functional categories related to other stress response. Profound transcriptional reprogramming during heat stress involves extensive regulation of transcription that affects a large part of the whole transcriptome (Zeller et al., 2009; Zou et al., 2011a).

The differential expression of 243 TFs among the 10 ecotypes indicates a complex level of transcriptional regulation during the exposure of plants to heat stress. Due to the lack of experimentally validated transcriptional regulatory information in *A. thaliana*, an *in-silico* transcript regulatory network model during cellular responses to heat stress in *A. thaliana* was constructed using the homogeneous gene expression data. The predicted activities of the heat regulated TFs showed significant variations among 10 ecotypes (Figure 4). The observed differential regulatory activities among the heat regulated TFs might contribute to high temperature acclimation of the specific ecotypes. Swindell et al. (2007) reported that multiple stress treatments interact with HSF and HSP response pathways to varying extents, suggesting that there is a basis of cross-tolerance in plant species through a shock response network. Expression of HSPs confers heat stress tolerance in plants that leads to improved photosynthesis, assimilate partitioning, water and nutrient use efficiency, and membrane stability (Wahid et al., 2007). The function of HSPs in enhancing stress tolerance may vary among genotypes and also depends on the nature of the stress imposed upon the cell. Such quantitative variation in the gene expression among the *Hsp* genes in the 10 ecotypes is clearly visible from Figure 3. Heat stress leads to direct denaturation of cellular proteins. Earlier, some *in vitro* data indicated that HSPs acted as molecular chaperones to prevent thermal aggregation of proteins by binding non-native intermediates which could then be refolded in an ATP-dependent fashion by other chaperones (Lee and Vierling, 2000). Therefore, the molecular chaperone activity of the HSPs may contribute to high temperature tolerance via prevention of protein misfolding and removal of non-native aggregations.

The 203 differentially regulated transposable elements (TEs) among the 10 ecotypes may play an important role in genome adapting to local climatic temperatures (Fedoroff, 2012). In a recent review, (Lisch, 2013) summarize the impact of stress activated retrotransposons on genome evolution in plants. Natural populations can show diverse responses when exposed to adverse environmental conditions because of their genetic variation as well as because of their epigenetic variations. Only a few studies have reported that stress responses in plants affect epigenetic regulation and require specific epigenetic regulators (Chinnusamy and Zhu, 2009). For example, UV, cold, and heat stress result in the reactivation of silent transgenes and endogenous transposable elements, although without reductions in DNA methylation and repressive histone marks (Grativol et al., 2012; Popova et al., 2013). Pecinka et al. (2010) showed that several repetitive elements of *A. thaliana* are under epigenetic regulation by transcriptional gene silencing at ambient temperatures and become activated by prolonged heat stress. A change in the epigenetic state of TEs by heat stress might also contribute to regulatory activities for adjacent genes. Recently, Wang et al. (2013) demonstrated that both TE sequence polymorphisms and the presence of linked TEs are positively correlated with intraspecific variation in gene expression. Some of the differentially regulated TEs in our heat experiments may therefore, be potentially interesting targets to explore diversity of heat stress responses among different ecotypes. Further targeted experiments in this direction can explore the molecular details of any potential role of these TEs on genomic adaptation of the ecotypes to their local environment.

## Materials and methods

### Microarray data

We have considered all the heat stress microarray experiments conducted on 10 ecotypes during the ERA-PG Multi-stress project (Rasmussen et al., 2013) to explore genome-scale transcriptome response signatures of *A. thaliana* during heat stress (microarray data available at GEO with the accession **GSE41935)**. All experiments of ERA-PG Multistress project were performed in environmentally controlled rooms at the plant growth facilities at RISØ DTU National Laboratory for Sustainable Energy (Roskilde, Denmark). A pilot study using wild type Col and L*er* plants was set up to find the appropriate conditions at sub-lethal doses (Rasmussen et al., 2013). These initial observations indicated that an optimal time before the onset of a phenotypic response (e.g., wilting, dehydration) while avoiding tissue damage was 3 h. 10 *A. thaliana* wild ecotypes (Table 1) were grown in soil under long day photoperiod and 24°C in a greenhouse setting for one generation to amplify homogeneous seed for all different genotypes. The seeds were then sown into trays and grown in a Conviron growth chamber (Winnipeg, Manitoba, Canada) under a 12h/12h photoperiod, 24°C and standard *A. thaliana* growth conditions. 3 week-old plants were then placed for 3 h in the environmentally controlled growth rooms that were preset to heat stress conditions (38°C). Triplicated (biological) trays with the wild type controls were subject to the heat stress. After the stress treatments, leaf samples were collected and promptly frozen in liquid nitrogen for subsequent microarray experiments.

### Statistical analysis of the data

Resulting data from the microarray experiments was pre-processed using the RMA (Irizarry et al., 2003) implementation in the oligo package (Carvalho and Irizarry, 2010) in R programming platform (R Core Team, 2012). Gene annotation was acquired from TAIR10 (Lamesch et al., 2012) using the BioMart data mining tool (Guberman et al., 2011). Differentially expressed genes between control and treated plants were identified using *t*-test (*p* < 0.01). Genotype specific responses to stress were identified by the interaction effect from a Two-Way ANOVA (Kerr et al., 2000; Cui and Churchill, 2003) of the genotype and treatment effect (*p* < 0.01). The union of stress responsive genes were further used for network-based analysis. Heat maps were plotted using TM4 microarray software suite (Saeed et al., 2006).

### Gene set enrichment analysis (GSEA)

The Biological Networks Gene Ontology (BiNGO) tool (Maere et al., 2005), an open-source Java tool, was used to determine Gene Ontology (GO) terms (Ashburner et al., 2000) that were significantly overrepresented in our differentially regulated gene lists (*p*-values were Bonferroni corrected).

### Network component analysis and network reconstruction

Network component analysis is a computational method for reconstructing the hidden regulatory signals (TFAs-Transcription Factor Activities) from gene expression data with known connectivity in terms of matrix decomposition (Liao et al., 2003; Galbraith et al., 2006). The algorithm for NCA analysis is implemented in MATLAB by Liao and colleagues and is online for download (www.ee.ucla.edu/~riccardo/NCA/nca.html). With NCA as a reconstruction method, we predicted significant TFs and connectivity strength on target genes and TFAs of TFs.

## Author contributions

Atle M. Bones and John Mundy conceived the Multi-Stress project. Pankaj Barah developed the concept of the current manuscript, performed bioinformatics analyses and drafted the manuscript. Naresh D. Jayavelu performed the NCA analysis. John Mundy led the ERA-NET PG Multi-Stress project, his laboratory generated all sample RNA/cDNAs. Atle M. Bones coordinated the overall development of the manuscript. All authors contributed toward improvement of the manuscript and have read and approved the manuscript.

## Conflict of interest statement

The authors declare that the research was conducted in the absence of any commercial or financial relationships that could be construed as a potential conflict of interest.

## Supplementary material

Table S1 **List of differentially regulated transcripts during heat stress. (A)** List of all the transcripts whose expressions were altered, during heat stress in the 10 ecotypes. Both *p*-values and log2 fold change values have been included along with short annotations. **(B)** List of 244 transcription factors (TFs) whose expressions were altered, during heat stress in the 10 ecotypes. Both *p*-values and log2 fold change values have been included. **(C)** List of 203 transposable elements (TEs) whose expressions were altered, during heat stress in the 10 ecotypes. Both *p*-values and log2 fold change values have been included.

Table S2 **Results of Gene Set Enrichment Analysis (GSEA) using BinGO software**.

Table S3 **List of Heat Shock Protein coding genes (*Hsp*s)**. List of all the 145 *Hsps* with corresponding *p*-values and log2 fold change values during the heat stress treatment in 10 ecotypes.

Table S4 **TF-TG regulatory bipartite connections predicted using NCA algorithm**. TF-TG regulatory bipartite connections predicted using NCA algorithm based on their expression profiles, using Pearson correlation coefficient threshold (PCC) ≥0.80. Annotations and functional roles (TF/TG) are also provided.
